# Intestinal intussusception in Peutz Jeghers syndrome: A case report

**DOI:** 10.1016/j.amsu.2020.04.013

**Published:** 2020-05-04

**Authors:** Salsabil Nasri, Tarak Kellil, Mohamed Ali Chaouech, Khadija Zouari

**Affiliations:** Department of Digestive Surgery, Fattouma Bourguiba University Hospital, Monastir, Tunisia

**Keywords:** Gastrointestinal intussusception, Peutz jeghers polyposis, Resection, Case report

## Abstract

Hamartomatous polyposis is a rare cause of intussusception in adults. But this complication is the most frequent for patient with Peutz Jeghers syndrome. Small bowel screening is recommended for those patients in order to prevent emergency repetitive surgeries.

We report here the case of a 20-year-old patient with confirmed Peutz Jeghers syndrome since eight years for whom a scheduled laparotomy was indicated. Asymptomatic intestinal intussusception was discovered intraoperatively. The patient was treated successfully with enterectomy and side to side anastomosis. Postoperative course was uneventful. Regular assessment as recommended for those patients is performed.

Gastrointestinal intussusception in adults is rare and is often diagnosed preoperatively in a context of bowel obstruction. In the case of our patient, intussusception was diagnosed intraoperatively. This fact confirms the necessity of well-timed polypectomy in order to prevent this complication and the risk of extended resection in patients who are exposed to short gut syndrome by requiring iterative resections.

## Highlights

• Intestinal intussusception is rare in adults.
• Intussusception is the most frequent complication of hamartomatous polyposis in Peutz Jeghers syndrome.
• Regular screening for those patients is necessary inorder to perform prophylactic polypectomy and to avoid emergency laparotomy.
• In our case intestinal intussusception was discovered intraoperatively, suggesting that this complication is unavoidable.
• This case highlights that economic resection for Peutz Jeghers patients is necessary to prevent short gut syndrome.

## 1 Introduction

Acute intestinal intussusception in adults is rare. It represents 5% of all intussusceptions and accounts for only 1–5% of intestinal obstruction in adults [1].

We report here the case of an asymptomatic intestinal intussusception discovered intraoperatively in a patient with a hamartomatous polyposis of Peutz Jeghers for whom a scheduled laparotomy was indicated. Our case is exposed and written according to the SCARE 2018 guidelines [2]. This case report illustrates the importance of regular assessment for patients with Peutz Jeghers syndrome in order to prevent complications. Well-timed polypectomy may avoid emergency surgery and extended resection in patients with high risk of short gut syndrome.

## 2 Case presentation

This is the case of a 20-year-old patient who has been known to have Peutz Jeghers syndrome since 2011. The diagnosis was based on the presence of periorificial lentiginosis and polyps of the upper intestinal tube. Their hamartomatous nature was confirmed by biopsies. She was operated on twice: in 2011 and in 2015 for large polyps of the proximal jejunum and she has had a resection anastomosis. Otherwise, there was no familial history of polyposis. She was regularly monitored as recommended.

**Fig. 1:**
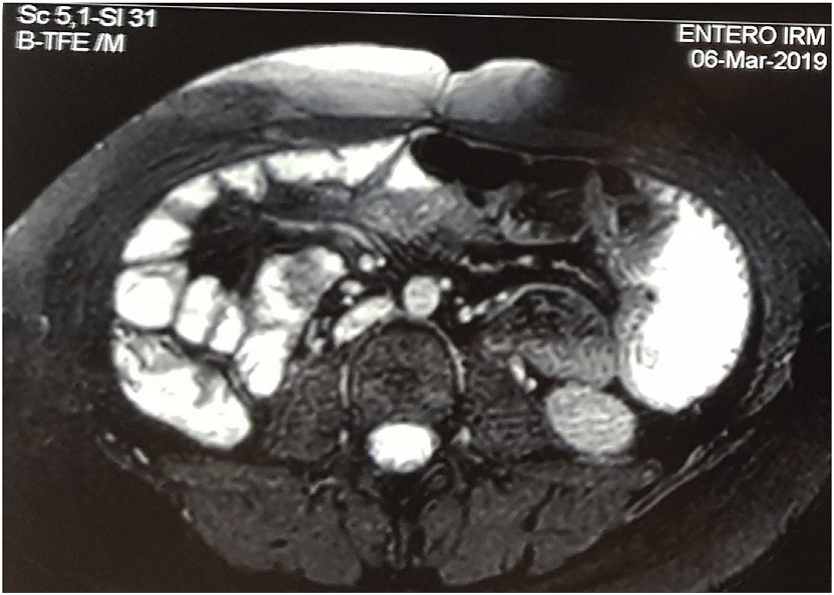
Entero-MRI showing 2 polyps in the right iliac fossa.

In March 2019, an entero-MRI performed as part of its regular monitoring assessment showed the presence of two pedunculated polyps, the largest of which measures 1.7 cm. They were located in the right flank with T1 hyposignal, T2 isosignal, homogeneously enhanced after injection of gadolinium. There was no sign of obstruction (Fig. 1). In view of the risk of acute complications, and the risk of malignant transformation, a surgical polypectomy was indicated. Endoscopic resection was not possible in this case.

**Fig. 2:**
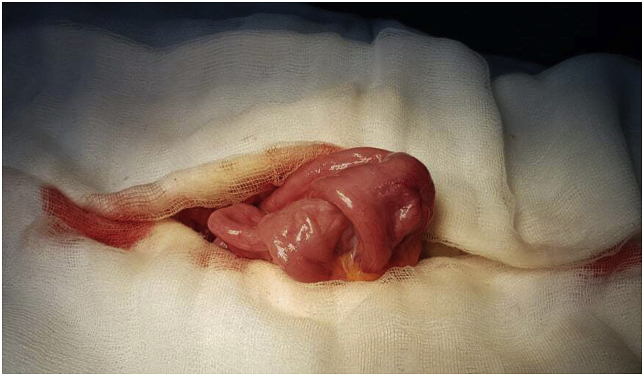
Intraoperative gastrointestinal intussusception.

**Fig. 3:**
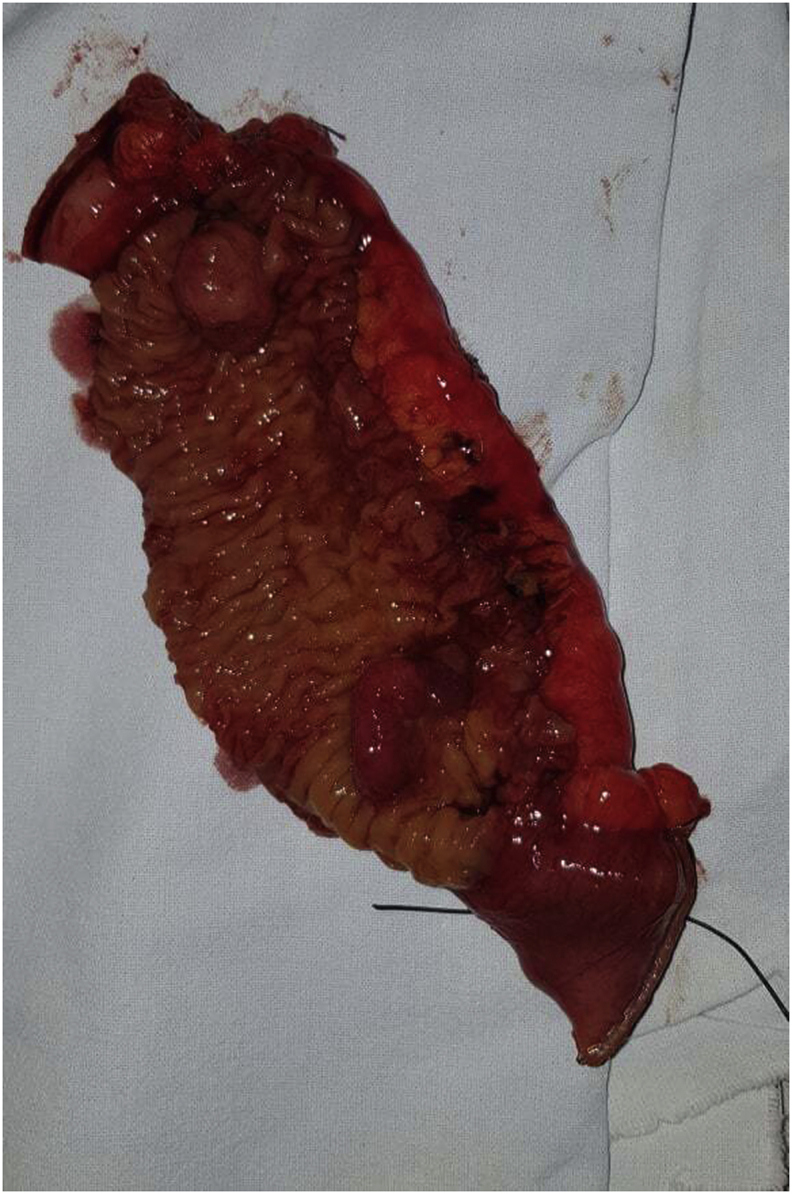
The specimen with 2 pedunculated polyps.

A scheduled laparotomy was performed by a university assistant in general surgery. Intraoperatively, we discovered the presence jejunojejunal intussusception, located at 1 m of the ileocaecal junction (Fig. 2). Manual reduction revealed the presence of two pedunculated polyps measuring approximately 2 cm. In addition, there was no intestinal distension and the palpation of the rest of the small bowel did not find other polyps. An economic resection of 20 cm of the small bowel carrying away the 2 polyps was performed with side to side enteric anastomosis (Fig. 3). The postoperative course was uneventful. The pathological examination of the specimen confirmed the hamartomatous nature of these polyps, without signs of malignant transformation. Regular assessment of our patient is maintained as recommended and she is adhering for her screening program.

## 3 Discussion

Peutz-Jeghers syndrome (PJS) was first described by Peutz in 1921 and Jeghers in 1944 and 1949 [3]. It is a rare entity. Its prevalence is around 1 in 100,000 people [4].

It is characterized by autosomal dominant inheritance, hamartomatous polyps of the gastrointestinal tract, characteristic mucocutaneous pigmentation and multiple neoplasms. This syndrome is seen in both male and female patients with no racial predominance [5]. It is usually diagnosed during the first two decades. Inheritance of the syndrome is autosomal dominant with incomplete penetrance, with some cases arising from spontaneous mutations. Mutations to the serine/threonine kinase 11 (STK11) tumour suppressor gene on chromosome 19p13.3 have been shown to cause Peutz-Jeghers syndrome [6].

The World Health Organization has laid the following criteria allowing clinical diagnosis of Peutz–Jeghers syndrome: three or more histologically confirmed Peutz–Jeghers polyps, any number of Peutz–Jeghers polyps with family history of Peutz–Jeghers syndrome, characteristic mucocutaneous pigmentation with a family history of Peutz–Jeghers syndrome, any number of Peutz–Jeghers polyps and characteristic mucocutaneous pigmentation [7]. Pigmentations usually appear in the first year of life but in adulthood may fade and in some cases even disappear. Pigmentation on the lips and buccal mucosa are an essential feature, playing an important role in the early diagnosis [4,8]. Polyps found in PJS are commonly present in adolescence and early adulthood. Over 90% of affected individuals will develop polyps in the small intestine during their lifetime. The incidence within the small intestine is greatest in the jejunum and progressively decreases in the ileum and duodenum. Involvement of the colon (53% of patients), stomach (49%), and rectum (32%) is also seen [4]. Extra-intestinal polyps have also been reported in the gallbladder, the ureter, respiratory tract and on the tonsils [4]. Patients usually present in the first decade due to polyp-related complications like abdominal pain, bowel obstruction, intussusception or occult gastrointestinal bleeding [9].

In our case, the diagnosis was retained according to the fourth WHO's criteria and our patient presented recurrent intestinal intussusception at three times.

PJS patients are markedly at risk for several malignancies, in particular gastrointestinal cancers and breast cancer. The reported lifetime risk for any cancer varied between 37 and 93% at the age of 60–70 years [10]. The most common cancers reported in the literature are gastrointestinal, gynecological, colorectal, pancreatic, and lung cancers [10].

Patients with affirmed Peutz Jeghers syndrome must have close and regular surveillance to prevent complications, so as to improve their outcome.

Periodic surveillance and removal of larger polyps aim to reduce the likelihood of complications in Peutz-Jeghers syndrome. Hence, as suggested by the American college of gastroenterology clinical guidelines, patients should undergo an annual complete blood cell count, as well as an annual physical examination that includes evaluation of the breasts, abdomen, pelvis, and testes. Lifelong cancer surveillance is advocated. Surveillance for gastric and small-bowel polyposis should begin at age 8–10 years and continue at 2–3-year intervals indefinitely [11]. If few or no polyps are found at the initial examination, screening should commence again at the age of 18. Small bowel screening could be performed using video capsule endoscopy (VCE)). Magnetic resonance enterography (MRE) is a reasonable alternative but it could underestimate polyps < 1.5 cm [8,9,11].

Intestinal intussusception has been observed in 47%–69% of adult patients with Peutz–Jeghers syndrome [12]. In the cohort of Wang and al, the diagnosis of intestinal intussusception was preoperative, only 1 case was discovered intraoperatively [12]. The particularity of our observation is that the intussusception was asymptomatic and discovered intraoperatively, suggesting that this complication is unavoidable and may be iterative without symptoms.

Polyp removal is the standard therapy to prevent complications. In fact, well-timed polypectomy may obviate the need for repeated urgent operations and extensive small bowel resections leading to short bowel syndrome. For many decades, it was based on laparotomy and bowel resection to remove symptomatic gastrointestinal polyps. However, some patients require multiple surgical resections, which can lead to short gut syndrome. A “clean sweep” is a combined endoscopic and surgical procedure which is actually recommended for the management of small-bowel polyps in Peutz Jeghers syndrome. The aim of this approach is to help to eliminate gastrointestinal symptoms and prevent or postpone repeat abdominal surgeries. We didn't practice this procedure due to the lack of ressources intraoperatively. We were limited to the manual palpation which didn't find other lesions.

## 4 Conclusion

Small bowel screening for patient with Peutz Jeghers syndrome is crucial since it helps to detect polyps earlier and prevent emergency surgery for intestinal intussusception. It offers the possibility of endoscopic resection, and economic enteric resections in order to prevent short gut syndrome.

## Patient consent

The patient accepted the publication of his case report**.**

## Ethical approval

Exemption from ethnical approval.

## Sources of funding

This is not applicable for our manuscript.

## Author contribution

Salsabil NASRI: data analysis and writing the paper.

Tarek KELLIL: data collection.

Mohamed Ali CHAOUECH: bibliography.

Khadija ZOUARI: revision.

## Registration of Research Studies

This is not applicable to our case report.

## Guarantor

Tarek KELLIL

## Consent

The patient's consent for the publication was solicited.

## Declaration of competing interest

Authors declare that there is no conflict of interest.
